# Role of probiotic extracellular vesicles in inter-kingdom communication and current technical limitations in advancing their therapeutic utility

**DOI:** 10.20517/evcna.2024.39

**Published:** 2024-09-13

**Authors:** Rahul Sanwlani, Kyle Bramich, Suresh Mathivanan

**Affiliations:** Department of Biochemistry and Chemistry, La Trobe Institute for Molecular Science, Melbourne 3086, Victoria, Australia.; ^#^Authors contributed equally.

**Keywords:** Extracellular vesicles, probiotic extracellular vesicles, bacterial extracellular vesicles, outer membrane vesicles, membrane vesicles, species crosstalk, inter-kingdom communication

## Abstract

Diverse functions of probiotic extracellular vesicles (EVs) have been extensively studied over the past decade, proposing their role in inter-kingdom communication. Studies have explored their therapeutic role in pathophysiological processes ranging from cancer, immunoregulation, and ulcerative colitis to stress-induced depression. These studies have highlighted the significant and novel potential of probiotic EVs for therapeutic applications, offering immense promise in addressing several unmet clinical needs. Additionally, probiotic EVs are being explored as vehicles for targeted delivery approaches. However, the realization of clinical utility of probiotic EVs is hindered by several knowledge gaps, pitfalls, limitations, and challenges, which impede their wider acceptance by the scientific community. Among these, limited knowledge of EV biogenesis, markers and regulators in bacteria, variations in cargo due to culture conditions or EV isolation method, and lack of proper understanding of gut uptake and demonstration of *in vivo* effect are some important issues. This review aims to summarize the diverse roles of probiotic EVs in health and disease conditions. More importantly, it discusses the significant knowledge gaps and limitations that stand in the way of the therapeutic utility of probiotic EVs. Furthermore, the importance of addressing these gaps and limitations with technical advances such as rigorous omics has been discussed.

## INTRODUCTION

Probiotics are live microorganisms that confer beneficial effects on the host, typically by mediating changes to the gut and intestinal microbiota^[1]^. Over the last few decades, their popularity and consumption have surged due to reported benefits^[2]^. Additionally, probiotics have been shown to positively impact interactions within the gut-brain axis^[3]^. Changes in microbiota composition have also been associated with various diseases, including inflammatory bowel disease (IBD), obesity, and irritable bowel syndrome (IBS)^[4-6]^. However, there are conflicting reports on the clinical efficacy of supplemented probiotics based on the pre-microbiota of patients^[7]^, as well as in murine models of colitis^[8]^. To address these discrepancies, future research must further investigate the complex factors influencing individual responses, such as gut microbiota composition, diet, and age. Regardless, the implication of altered microbiota during disease progression has spurred interest in exploring the therapeutic potential of probiotic strains. Recent studies have demonstrated that the administration of specific probiotic species can elicit beneficial responses in models of colitis, IBS, and colorectal tumorigenesis^[9-11]^. Therefore, identifying the specific factors most responsible for these observed changes has become an area of intrigue. Given the importance of intercellular communication in maintaining microbiome symbiosis, probiotic extracellular vesicles (EVs) are thought to play a role in mediating these benefits^[12]^. The well-established role of EVs in intercellular communication and their reported benefits in complex disease models make them a promising area of study. Thus, recent investigations have expanded to explore the role of probiotic EVs in a wider range of functions by influencing the surrounding gut microbiota^[13]^.

EVs have been generally defined as lipid bilayer structures that contain membrane proteins and a diverse range of cargo, including proteins, metabolites, DNA, and RNA^[14]^. Most EV subtypes range in size from 30 nm to 10 µm and are typically classified based on distinct characteristics such as biogenesis, size, content, and cell of origin^[15]^. Gram-positive and Gram-negative bacteria release distinct types of EVs, generally smaller than 500 nm, termed membrane vesicles (MVs) and outer membrane vesicles (OMVs), respectively^[16,17]^. MVs consist of a lipid bilayer enclosing cytoplasmic material, while OMVs are enclosed by the outer membrane and, therefore, also contain periplasmic material in addition to cytoplasmic contents. Additionally, the overall surface and embedded membrane proteins are distinct between these two types of EVs^[18]^. Despite this, the mechanisms and regulation of EV biogenesis in bacteria remain poorly understood^[19]^. Consequently, the lack of reliable EV markers hinders thorough characterization in the bacterial EV field^[20]^. Notably, it has been observed that the DNA/RNA ratio in probiotic-derived EVs can vary significantly among different probiotic species^[21]^. Identifying bacterial EV markers will likely lead to improved purity and characterization, potentially enabling researchers to pinpoint specific subtypes responsible for particular effects. Therefore, in this review, the term “probiotic EVs” will be employed to refer to vesicles from various sources, given the current lack of complete understanding and characterization.

Although probiotic EVs are biochemically lipid bilayers encapsulating a diverse range of biomolecules, they may possess several unique attributes that distinguish them from other EVs. A recent study characterized EVs from *Lactobacillus* species, revealing an enrichment of proteins with a serine-rich motif^[22]^. Additionally, EVs secreted by *Lactobacillus* species were found to be rich in proteins and enzymes involved in metabolic processes such as gluconeogenesis, glycolysis, amino acid and carbohydrate metabolism^[22-24]^. This enrichment suggests that probiotic bacteria may employ EVs as conduits to convey systemic benefits or regulate pathophysiological processes. For instance, studies have shown that *Lactobacillus* species secrete EVs enriched with antimicrobials, proteases, and proteins p40 and p75, which have been identified as mediators of probiotic effects^[22,25-27]^. Further research could uncover additional properties of probiotic EVs that differentiate them from other EVs.

## THE PATHOPHYSIOLOGICAL ROLE OF PROBIOTIC EVs THROUGH INTER-KINGDOM AND SPECIES CROSSTALK

**Figure 1:**
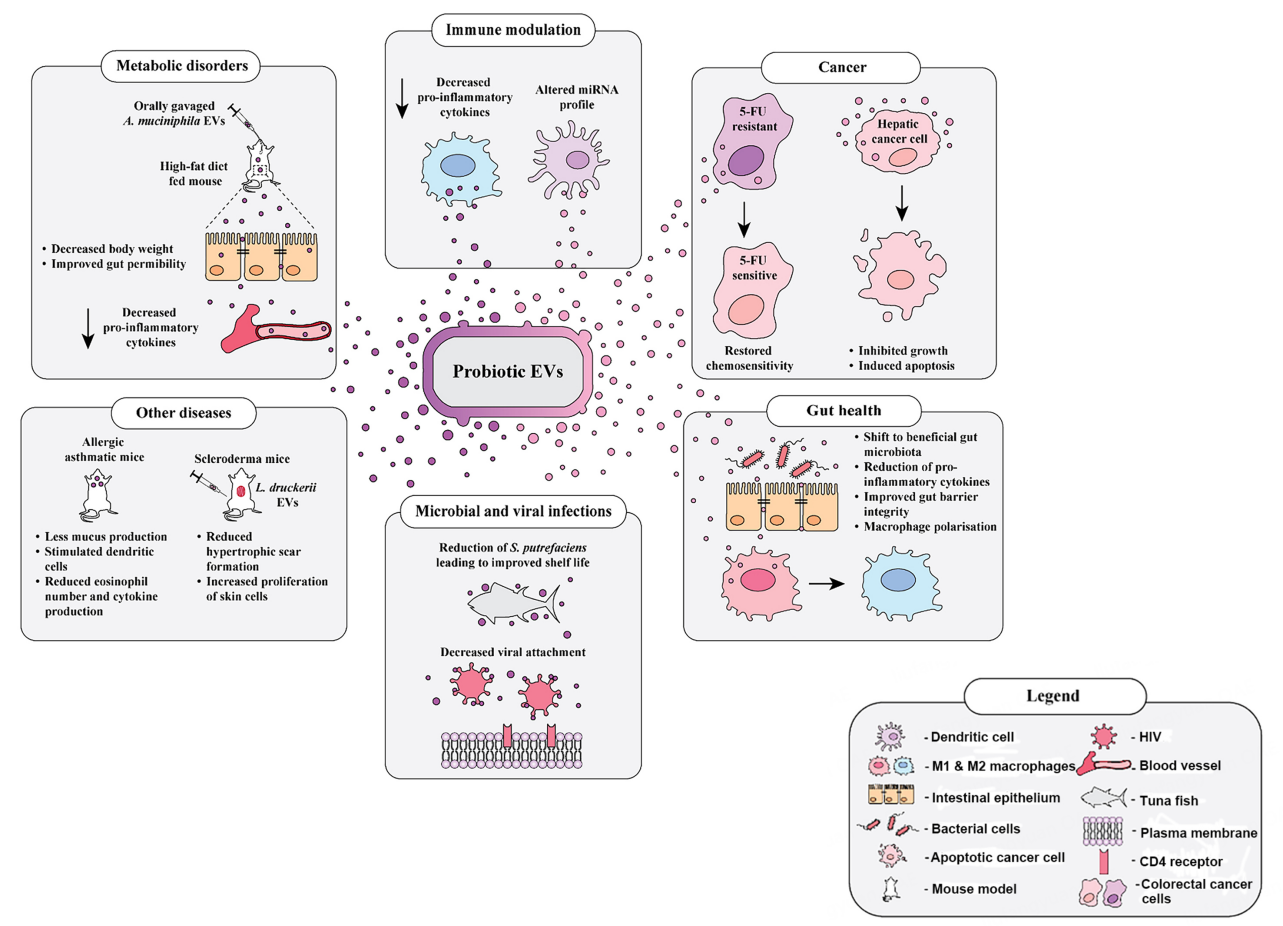
Implicated beneficial effects of probiotic extracellular vesicles in pathophysiological conditions. Schematic representation of the implicated roles that probiotic-derived EVs play in a plethora of pathophysiological processes. Several studies so far have demonstrated the potential of probiotic EVs in modulating the immune system and alleviating pathological conditions such as IBD, diabetes, and ulcerative colitis. The suggested health benefits of probiotic EVs range from improving gut health to inducing chemosensitivity and apoptosis in resistant cancer cells. Their role in several other diseases such as asthma, metabolic disorders including diabetes and obesity and their antimicrobial properties have been investigated in several studies. Table 1 provides a detailed summary of studies where the role of probiotic EVs in pathophysiology and disease alleviation has been suggested. IBD: Inflammatory bowel disease; EVs: extracellular vesicles; HIV: human immunodeficiency virus.

Probiotics have been shown to elicit a wide range of beneficial effects in various disease states, including cancer, IBS, and upper respiratory tract infections^[28-30]^. However, the underlying mechanisms of how probiotics mediate these effects remain poorly understood. Recently, EVs derived from probiotic strains have been implicated in important functional roles in pathophysiology [Figure 1]. Several studies have presented evidence that probiotic EVs laden with a variety of functional cargos may be responsible for initiating or mediating crucial signaling events that can alleviate disease conditions [Table 1].

**Table 1:** Role of probiotic EVs in pathophysiology

**Source of EVs**	**Function observed**	**Model**	**Functional cargo**	**Reference**
**Antimicrobial activity**				
*Lactobacillus acidophilus*	Bacteriocin delivery and antimicrobial activity	*In vitro*	Not stated	[26]
*Bacteroides thetaiotaomicron, Enterobacter cloacae, Lactobacillus acidophilus*	Altered EV cargo, biogenesis, size and quantity	*In vitro*	Not stated	[31]
*Lactobacillus paracasei*	PEGylated MoS2-ZnO containing EVs used as antimicrobial agents to restrict the growth of *Staphylococcus aureus*	*In vitro*	Not stated	[32]
*Lactobacillus plantarum* WCFS1	EVs promoted expression of host defense genes and inhibited growth of vancomycin-resistant *Enterococcus faecium*	*In vivo*	Not stated	[33]
*Lactobacillus plantarum*	EVs reduced bacterial levels of *Shewanella putrefaciens* and maintained better food quality, increasing shelf life	*Ex vivo*	Not stated	[34]
*Lactobacillus crispatus* BC5*, Lactobacillus gasseri* BC12	Probiotic EVs maintained healthy vaginal homeostasis by supporting colonization of beneficial bacterial species and preventing attachment of opportunistic pathogens	*In vitro*	Not stated	[35]
*Lactobacillus plantarum*	Probiotic EVs displayed protective effects in atopic dermatitis induced by *Staphylococcus aureus* EVs	*In vivo*	Not stated	[36]
**Antiviral activity**				
*Staphylococcus aureus, Gardnerella vaginalis, Enterococcus faecium, Enterococcus faecalis*	EVs prevented HIV-1 infection in tissues *ex vivo* by preventing virus-cell receptor interaction	*Ex vivo*	Not stated	[37]
*Lactobacillus crispatus* BC3 and *Lactobacillus gasseri* BC12	Probiotic EVs prevented HIV-1 infection in cervico-vaginal and tonsillar tissues *ex vivo*	*Ex vivo*	Not stated	[38]
**Immunomodulatory effects**				
*Lactobacillus sakei subsp. sakei* NBRC 15893	Enhanced IgA production in Peyer’s patch cells in response to membrane vesicles	*In vivo*	Not stated	[39,40]
*Lactobacillus rhamnosus* GG and *Lactobacillus reuteri* DSM 17938	Lactobacilli MVs dampen pro-inflammatory cytokine responses in a monocyte-dependent manner.	*In vitro*	Not stated	[41]
*Lactobacillus plantarum*	Regulation of pro and anti-inflammatory responses; innate and acquired. Enhanced IgA production in Peyer’s patch cells	*In vitro*	N-acylated peptides from lipoprotein19180	[42]
*Escherichia coli* Nissle 1917	*E. coli* Nissle 1917 enhanced secretion levels of pro- and anti-inflammatory cytokines in monocyte-derived dendritic cells	*In vitro*	Not stated	[43]
*Lactobacillus plantarum*	EVs promoted differentiation of human monocytic THP1 cells toward an anti-inflammatory M2 phenotype	*In vitro*	Not stated	[44]
*Lactobacillus reuteri* BBC3	EVs maintained intestinal immune homeostasis against LPS-induced inflammatory responses	*In vivo*	Not stated	[45]
*Lactobacillus casei*	EVs exhibited anti-inflammatory immunomodulatory effects in intestinal epithelial cells	*In vitro*	Not stated	[46]
*Bifidobacterium longum, Clostridium butyricum, Lactobacillus plantarum* WCFS1	EVs stimulated the innate immune system and exhibited adjuvant-like properties	*In vitro*	Not stated	[47]
*Bifidobacterium longum* and *Lactobacillus plantarum* WCFS1	Immune cells produced pro-inflammatory cytokines in response to EVs	*In vitro*	Not stated	[48]
*Lactobacillus strains (L. rhamnosus NutRes 1, L. plantarum* NutRes 8 and *L. caseï* CNCM I-1518) and *Bifidobacterium strains (B. longum* NutRes 266*, B. breve* NutRes 200 and *B. animalis* DN173010*)*	Effect of EVs (TLR2 activity and phagocytosis ability) characterized on immune cells	*In vitro*	Not stated	[49]
*Lactobacillus plantarum*	pH-based stimulation of bacterial cells led to secretion of EVs with enhanced anti-inflammatory properties	*In vitro*	Not stated	[50]
*Lactobacillus johnsonii* N6.2	EVs led to antibody and immune response generation	*In vivo*	Not stated	[51]
*Escherichia coli* Nissle 1917	EVs caused RAW 264.7 macrophages to have increased phagocytosis, proliferation, and shifted their phenotype to more anti-inflammatory	*In vitro*	Not stated	[52]
*Bifidobacterium longum*	EVs induced apoptosis of mast cells. IP injection of ESBP led to reduced diarrhea occurrence and abundance of mast cells	*In vivo*	ESBP	[53]
*Propionibacterium freudenreichii* CIRM-BIA 129	EVs exhibited anti-inflammatory effect by modulation of the NF-κB pathway in a dose-dependent manner, partly but not completely dependent on surface proteins	*In vitro*	Not stated	[54]
*Leuconostoc mesenteroides, Latilactobacillus curvatus*, and *Lactiplantibacillus plantarum*	EVs exerted anti-inflammatory effects on microglial cells and macrophages	*In vivo*	Not stated	[55]
*Escherichia coli* Nissle 1917	NOD1 signaling activated by EVs in intestinal epithelial cells	*In vitro*	Not stated	[56]
*Escherichia coli* Nissle 1917	EV-treated macrophages exhibited anti-inflammatory effects and increased anti-bacterial activity	*In vitro*	Not stated	[52]
*Lacticaseibacillus rhamnosus* JB-1	EVs led to immunomodulatory effects in dendritic cells mediated by TLR2	*In vitro*	Lipoteichoic acid	[57]
*Lactobacillus, Bifidobacterium*, and *Lactococcus*	Probiotic EV administration post hepatic surgery reduced adhesion molecule expression and immune cell invasion in liver and contributed to improved livery recovery	*In vivo*	Not stated	[58]
*Escherichia coli* Nissle 1917	EVs treatment led to an altered miRNA profile in dendritic cells	*In vitro*	Not stated	[59]
*Bacillus amyloliquefaciens* SC06	Probiotic EV-treated porcine intestinal epithelial cells shed EVs, which led to improved macrophage function	*In vitro*	Not stated	[60]
**Gut health**				
*Lactobacillus plantarum* Q7	EVs induced a shift in the gut microbiota to a more anti-inflammatory community, reduction of pro-inflammatory cytokine expression in colon tissue, and alleviated DSS-induced colitis symptoms	*In vivo*	Not stated	[61]
*Lactobacillus rhamnosus* GG	EVs modulate gut microbiota and attenuated inflammation and ulcerative colitis	*In vivo*	Not stated	[62]
*Lactobacillus paracasei*	EV-mediated anti-inflammatory effects maintained gut health in response to LPS treatment and provided protection against ulcerative colitis	*In vivo*	Not stated	[63]
*Clostridium butyricum*	EVs protected against DSS-induced colitis by regulating the repolarization of M2-macrophages and remodeling gut microbiota	*In vivo*	Not stated	[64]
*Lactobacillus kefirgranum* PRCC-1301	EVs exhibited anit-inflammatory effects by inhibiting the NF-κB pathway and improving intestinal barrier function	*In vivo*	Not stated	[65]
*Lactobacillus kefir* KCTC 3611*, L. kefiranofaciens* KCTC 5075, and *L. kefirgranum* KCTC 5086	EVs exhibited anti-inflammatory effects and alleviated IBD symptoms	*In vivo*	Not stated	[66]
*Lactobacillus reuteri* DSM-17938	EVs mediate gut motility effect	*In vivo*	Not stated	[67]
*Escherichia coli* Nissle 1917	Modulation of intestinal tight junctions that were disrupted via enteropathogenic *E. coli*. Specifically, by maintaining important tight junction proteins and preventing F-actin disorganization	*In vivo*	Not stated	[68]
*Clostridium butyricum*	EVs alleviated colitis symptoms, improved gut barrier integrity, and restored gut microbiota homeostasis	*In vivo*	Not stated	[69]
*Escherichia coli* Nissle 1917	EVs exhibited gut barrier protective effects in enteropathogenic *E. coli*-infected intestinal epithelial cells	*In vitro*	Not stated	[68]
*Clostridium butyricum*	EVs demonstrated anti-inflammatory effects, alleviated bacterial dysbiosis, reduced abundance of pathogens, improved gut barrier integrity, and regulated metabolism of gut microbiota in colitis mice	*In vivo*	Not stated	[70]
*Lactobacillus plantarum*	Engineered EVs loaded with fucoxanthin had beneficial effects in colitis mice as they polarized macrophages and alleviated colitis symptoms and bacterial dysbiosis	*In vivo*	Not stated	[71]
**Cancer**				
*Lactobacillus plantarum*	EVs restored chemosensitivity in 5-FU resistant colorectal cancer cells	*In vitro*	Not stated	[72]
*Lactobacillus rhamnosus* GG	EVs inhibited growth of colorectal cancer cells	*In vitro*	Not stated	[73]
*Lactobacillus rhamnosus* GG	EVs inhibited hepatic cancer cell growth and induced apoptosis	*In vitro*	Not stated	[74]
*Lacticaseibacillus paracasei*	EVs had an anti-tumor effect *in vitro* on colorectal cancer cell proliferation, invasion and migration and promoted apoptosis *in vitro* and *in vivo*	*In vivo*	Not stated	[75]
**Other diseases/pathophysiology**				
*Akkermansia muciniphila*	Administration of A.muciniphila EVs decreased weight, improved glucose tolerance, and enhanced tight junction function in a HFD-induced mouse model. This treatment also improved gut permeability in HFD mice	*In vivo*	Not stated	[76]
*Akkermansia muciniphila*	*A. muciniphila* EVs prevented gut dysbiosis in a high-fat diet-induced mouse model. They also reduced the levels of pro-inflammatory cytokines in plasma, reduced food intake, and decreased the expression of genes involved in lipid metabolism and inflammation	*In vivo*	Not stated	[77]
*Akkermansia muciniphila*	EVs ameliorated HFD-induced obesity, and improved the intestinal barrier integrity, inflammation, energy balance, and blood parameters	*In vivo*	Not stated	[78]
*Lactobacillus plantarum*	EVs inhibited neuron apoptosis and protected against ischemic brain injury	*In vivo*	Not stated	[79]
*Lactobacillus plantarum, Akkermansia muciniphila, Bacillus subtilis*	EV treatment led to anti-depressive effects and alleviated stress-induced depressive behavior in mice	*In vivo*	Not stated	[80]
*Lactobacillus plantarum*	EVs displayed anti-depressant activity by rescuing reduced expression of BDNF and inhibited stress-induced depressive-like behavior	*In vivo*	Not stated	[81]
*Lactobacillus animalis*	EVs prevented the development of glucocorticoid-induced ONFH	*In vivo*	Not stated	[82]
*Lactobacillus reuteri*	No effect of EVs on osteoclastogenesis	*In vitro*	Not stated	[83]
*Lactobacillus druckerii*	EVs inhibited hypertrophic scar fibrosis	*In vivo*	Not stated	[84]
*Lactobacillus plantarum* and *Lactobacillus casei*	EVs from both species were coupled with microparticles; these bacteriomimetics were then embedded into a hydrogel mixture; the resulting combination improved wound healing	*In vivo*	Not stated	[85]
*Lactobacillus plantarum*	EVs regulated extracellular matrix-related genes and suppressed wrinkle formation and pigmentation in women	*In vivo*	Not stated	[86]
*Lactobacillus casei* BL23	EGFR pathway stimulation by EVs	*In vitro*	P40 and P75	[87]
*Akkermansia muciniphila*	EVs caused the regression of hepatic stellate cell activation in a liver injury mouse model. They also ameliorated levels of inflammatory cytokines, improved liver histopathological damages, and reduced the expression of fibrosis biomarkers	*In vivo*	Not stated	[88]
*Akkermansia muciniphila*	Oral administration of *A. muciniphila* EVs improved intestinal integrity and anti-inflammatory responses in a mouse model of liver injury	*In vivo*	Not stated	[89]
*Lactococcus lactis*	EVs exhibited an immunoregulatory effect on airway inflammation in allergic asthma by activating dendritic cells	*In vivo*	Not stated	[90]
*Leuconostoc holzapfelii*	EVs promoted proliferation, migration and regulated cell cycle in human hair follicle dermal papilla cells	*In vitro*	Not stated	[91]
*Akkermansia muciniphila* and *Faecalibacterium prausnitzii*	Treatment with EVs led to increased serotonin production in intestinal epithelial cells	*In vitro*	Not stated	[92]

The table summarizes the role of probiotic EVs in facilitating crossspecies and inter-kingdom communication by mediating complex signaling events in the host organism. The source of EVs, functional effect, and the experimental model in which the effect was studied have been specified. EVs: Extracellular vesicles; HIV-1: human immunodeficiency virus 1; IgA: immunoglobulin A; TRL2: toll-like receptor 2; NF-κβ: nuclear factor kappa β; DSS: dextran sodium sulfate; LPS: lipopolysaccharide; HFD: high-fat diet; ONFH: osteonecrosis of the femoral head; EGFR: epidermal growth factor receptor; HFD:high-fat diet.

### The utility of probiotic EVs as potential antimicrobial and antiviral treatments

The current understanding that the administration of probiotic EVs can impart beneficial effects during disease has sparked interest in their potential role in cross-species and inter-kingdom communication. Recent research has highlighted the antimicrobial activity of EVs derived from probiotic sources^[34-36,38]^. Interestingly, it has been shown that EVs isolated from *L. plantarum* can reduce the levels of *S. putrefaciens*, a bacterium indicative of rotting fish^[34]^. Tuna fish stored with *L. plantarum* EVs had an increased shelf life and overall food quality^[34]^. In another recent study, the ability of bioengineered EVs from *L. paracasei* to target and restrict *S. aureus* growth was demonstrated, suggesting their utility as nano antibiotics^[32]^. These studies clearly highlight the potential of probiotic EVs in inter-kingdom communication. Thus, recent research has also attempted to explore the possible interactions between probiotic EVs and the host cells in various diseases and health conditions. For instance, *L. plantarum* EVs were able to modify host cell defenses, offering increased protection against antibiotic-resistant bacterial pathogens^[33]^. In this regard, a better understanding of probiotic EV cargo and the associated signaling with host cells may reveal avenues for improved treatment of various diseases. In the context of antiviral activity, one study exhibited that EVs originating from symbiotic vaginal *Lactobacilli* species had the ability to reduce human immunodeficiency virus-1 (HIV-1) infection^[38]^. Human vaginal and tonsillar tissues were infected with HIV-1 *ex vivo* and then treated with EVs derived from *L. crispatus* and *L. gasseri* strains. EV treatment provided protection against HIV-1 attachment and entry due to reduced exposure to envelope glycoprotein (Env), a viral envelope protein crucial for virus-host cell interaction.

### The role of probiotic EVs in various disease states and the associated immunomodulatory effects

**Figure 2:**
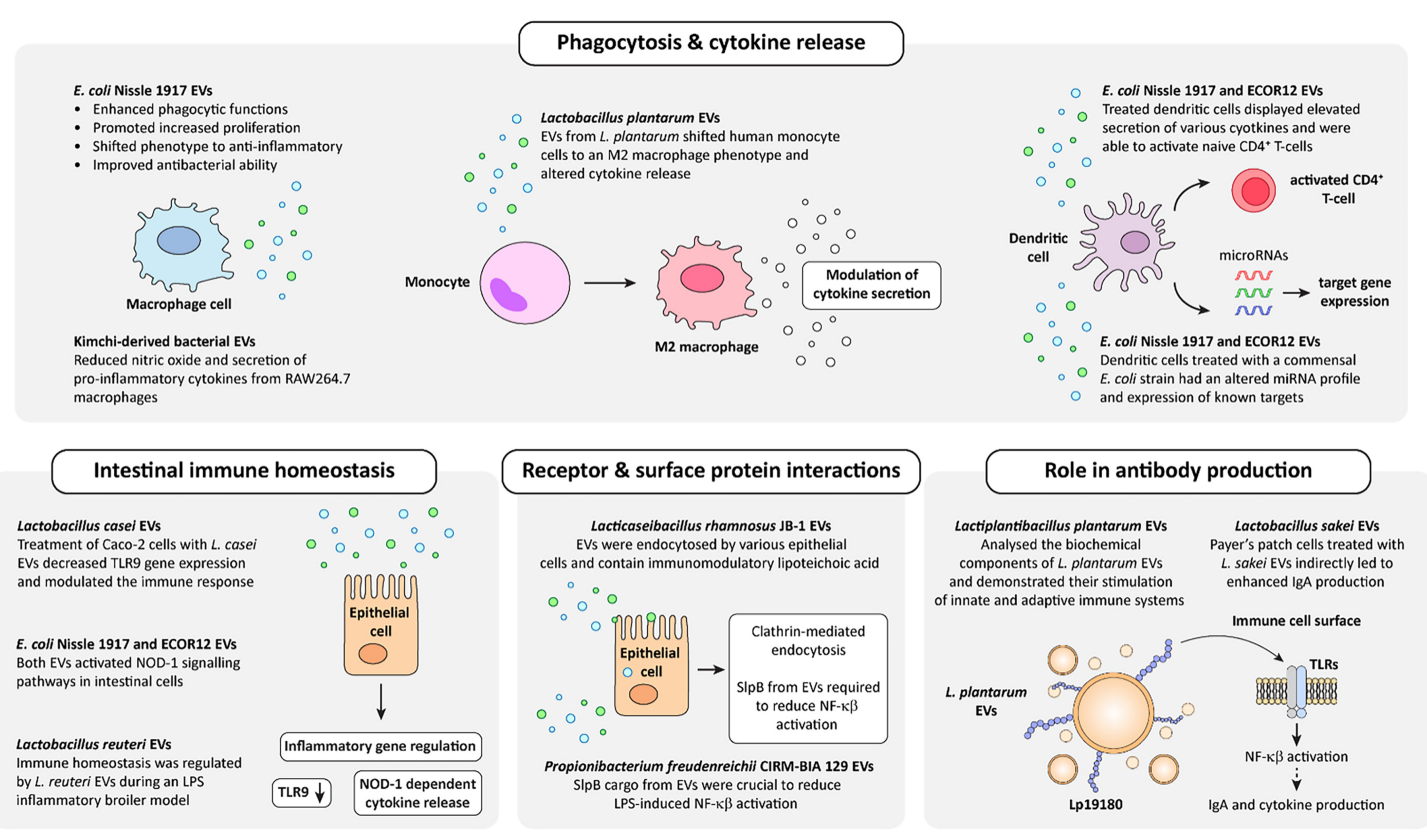
Immune regulatory effects of probiotic EVs. Schematic diagram depicting the various immunomodulatory roles of probiotic EVs, including alteration to cytokine release, activation of immune cells, surface interactions, and changes to antibody production. Table 1 provides a comprehensive summary of the literature suggesting a functional role for probiotic EVs in the immune setting. EVs: Extracellular vesicles; IgA: immunoglobulin A; TRL: toll-like receptor; LPS: lipopolysaccharide; NF-κβ: nuclear factor kappa β.

The immunomodulatory effects of probiotic EVs are their most well-characterized attribute so far [Figure 2]. Probiotic EVs from several bacterial genera, including *Lactobacillus*, *Escherichia*, and *Bifidobacterium*, have been associated with phenotypic changes in various immune cells^[44,49,59]^. EVs from *L. plantarum* promoted the differentiation of human leukemia monocytes toward an M2 phenotype, which is characterized by anti-inflammatory properties^[44]^. Moreover, probiotic EVs can also prime immune cells to mount a pro-inflammatory response *in vitro*. Dendritic cells treated with EVs derived from *E. coli* Nissle 1917, a widely studied probiotic, exhibited changes in their miRNA profile^[59]^. As a result, dendritic cells produced more Th1-type cytokines important for managing pathogens. Evidently, probiotic EVs can communicate with and influence a wide range of immune cells, stimulating beneficial phenotypic changes [Figure 2]. However, there have been several studies where their role in mediating a direct phenotype in disease conditions such as ulcerative colitis and IBD has been reported [Table 1].

The intestinal epithelium provides a crucial physical barrier while allowing intestinal epithelial cells (IECs) to participate in crosstalk between the gut microbiota and systemic immune system^[93]^. There is growing evidence to support the beneficial role of probiotic EVs in modulating gut health by communicating with IECs [Table 1]. Several species of *Lactobacilli* have been shown to promote an anti-inflammatory intestinal phenotype in an ulcerative colitis mouse model^[65,68,94]^, as well as reducing the expression of various pro-inflammatory cytokines. Additionally, there have been studies suggesting that alterations to DNA amount and specific immunostimulatory oligodeoxynucleotides within probiotic EVs can contribute to stronger immune activation^[21,95]^. Furthermore, EVs from probiotic sources have demonstrated the ability to improve gut barrier function. One study has shown that following a loss of gut barrier integrity induced by *E. coli*, EVs derived from *E. coli* Nissle 1917 improved gut barrier function^[68]^. Comparatively, this reduced permeability was associated with maintaining ZO-1 at tight junctions and conserving occludin and claudin-14 mRNA levels. Similarly, another study has revealed that *L. kefirgranum* EVs originating from kefir grain were able to conserve tight junctions and epithelial cell integrity^[65]^. Given this, the reported beneficial effects of probiotics EVs are not limited to solely immune modulation and gut health. Emerging evidence has demonstrated the ability of probiotic EVs to disturb detrimental signaling pathways involved in cancer, obesity, liver injury, and even depression^[75,76,80,88]^. A deeper understanding of the precise mechanisms and EV-associated proteins involved in pathogenic and host cell communication is required within the field of probiotic EVs. Furthermore, addressing the challenges and limitations surrounding probiotic EV application is paramount for quality future research. This improved knowledge may allow for therapeutic interventions in disease, potentially leading to prevention and/or improved treatment.

Findings in these preclinical studies present an exemplary case for the clinical utility of probiotic EVs in therapeutic applications. Several clinical studies recently have investigated the utility of pathogen-derived OMVs in designing vaccines against viral and bacterial infections^[96]^. Furthermore, there are clinical studies investigating the potential benefits of probiotics with promising preliminary outcomes^[97-99]^. However, to date, there are no clinical studies investigating the role of probiotic extracellular vesicles in combating infectious or non-infectious diseases. There are several technical limitations and challenges leading to knowledge gaps that have hindered the clinical utility of probiotic EVs.

## CHALLENGES AND LIMITATIONS TO EMPLOYING PROBIOTIC EVs IN CLINICAL APPLICATIONS

**Figure 3:**
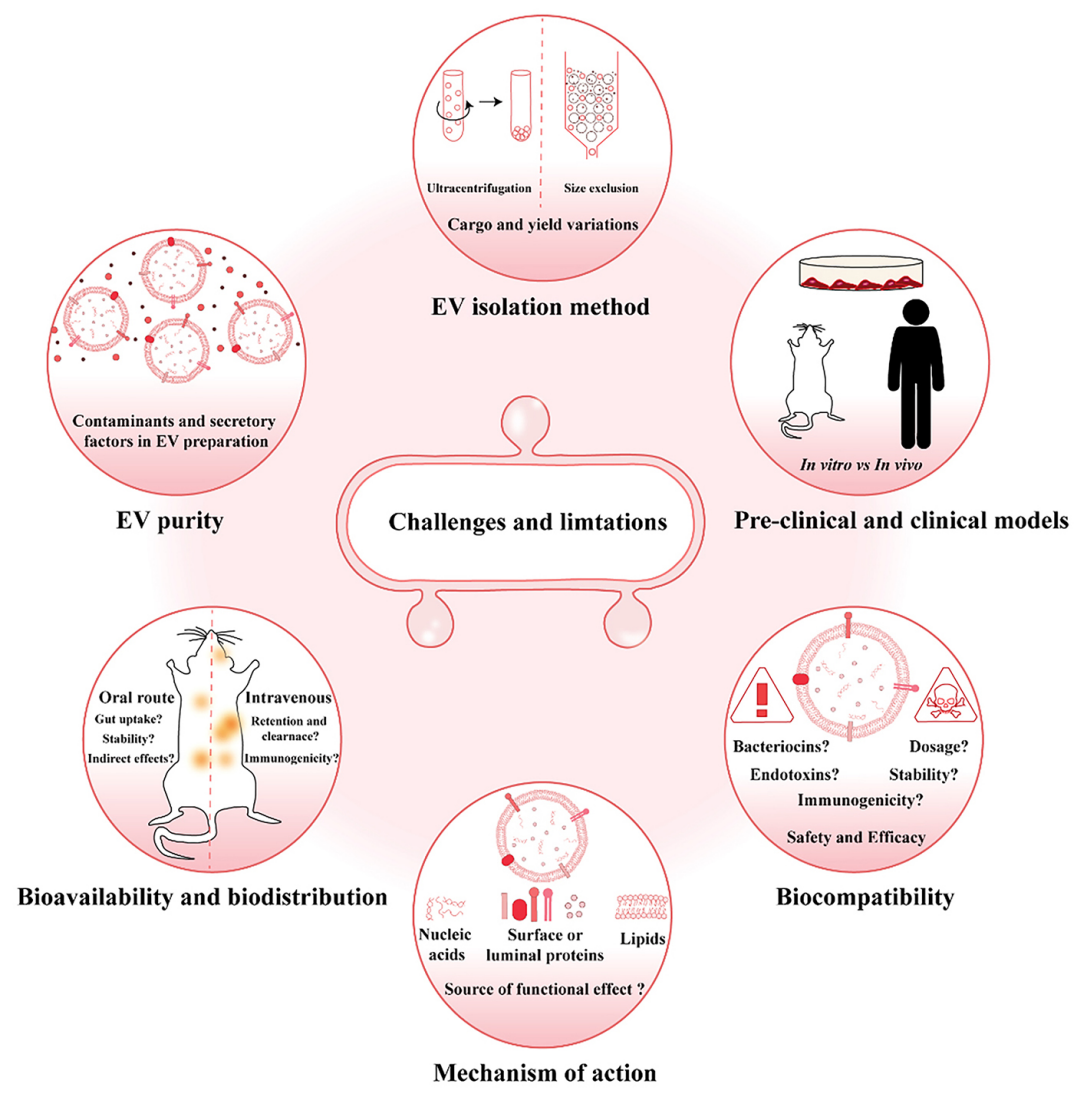
Current challenges and technical limitations hindering the therapeutic utility of probiotic EVs. Schematic illustration to depict challenges and limitations facing the utility of probiotic EVs in clinical applications. Several technical constraints such as EV cargo and yield variations due to different isolation methods, the presence of secretory factors and contaminants in the preparation, and the use of inappropriate preclinical models to study the effect and variation in phenotype based on the route of administration could prevent a detailed understanding and widespread acceptance of the role of probiotic EVs in pathophysiology. Additionally, current technical limitations prevent a better understanding of the EV cargo component responsible for the observed phenotypes. The annotated bibliography is shown in the Supplementary Materials. EVs: Extracellular vesicles.

EVs secreted by probiotic and commensal microbiota and derived from dietary sources have been proposed to be mediators of cross-species and inter-kingdom communication^[13,100,101]^. Their ability to signal in complex pathophysiological events enables them to dictate phenotype in a range of health and disease conditions [Table 1]. Bovine milk extracellular vesicles (MEV) and probiotic EVs have previously been examined for their stability and resilience to withstand harsh, degrading gut conditions, assessing their oral bioavailability and were able to reach peripheral tissues^[102-104]^. They were reported to withstand boiling temperatures and acidification, supporting their role as vehicles that sequester bioactive cargo in its native, functional state upon exposure to extreme conditions^[104]^. Similarly, in other studies, it has been reported that regular industrial processing such as heat treatment could harm the integrity and molecular composition of MEVs. However, the extent of this damage is unclear as MEVs were recovered in high abundance from milk despite the harsh processing and their surface markers could still be detected upon molecular analysis^[104-107]^. Furthermore, EVs from dietary sources or gut bacteria have been speculated to cross the gut barrier through either transendocytosis or paracellular translocation, entering systemic circulation and reaching peripheral tissues^[23,51,102,108-110]^. Although the aforementioned evidence supports the function of probiotic EVs in pathophysiology, they are not entirely convincing as several knowledge gaps persist, serving as hindrances to the application of probiotic EVs in therapy [Figure 3]^[111,112]^. Contradictory findings have led to varying schools of thought about the potential and utility of probiotic EVs, making it imperative to address these knowledge gaps, challenges, and limitations for further advancement of the field^[111]^.

### EV biogenesis mechanisms, cargo regulation and isolation

EV biogenesis and secretion is conserved across all domains of life, with this process having only recently been identified in bacteria^[19,113]^. Although during the last decade, there has been a notable rise in interest in deciphering functions of probiotic EVs in inter-kingdom communication [Table 1], several gaps in our understanding of EV biogenesis in bacteria still persist^[19]^. Recent research has revealed that bacterial EV secretion occurs not only through membrane blebbing but also via explosive lysis^[114,115]^. Furthermore, several internal and external factors that could regulate or stimulate bacterial EV biogenesis and secretion are still being explored^[19]^. Despite the limited research on MV biogenesis and its regulators^[19]^, it is known that variations in environmental factors such as nutrients, pH, or exposure to antibiotics can result in significant variations in EV yield, cargo, and function^[31,101,116,117]^. For instance, oxygen exposure and oxidative stress, as environmental factors, have been linked to changes in bacterial EV cargo^[118]^. *Lactobacillus* species in another study were observed to secrete EVs with slightly varying bacteriocin activity^[26]^. Therefore, environmental conditions may lead to variations in EV cargo both *in vivo* and *in vitro*, leading to varying phenotypic observations across studies. Moreover, co-cultivation of two different probiotic strains has been shown to elevate the bioactivity of EVs by increasing the secretion of interleukins from peripheral blood mononuclear cells^[119]^. Moreover, studies have highlighted the effect of EV isolation methods on its yield and cargo, demonstrating varying cargo and function in EVs isolated from the same source using different methods^[120,121]^. Evidently, improved knowledge of EV biogenesis, mechanisms at play, and regulators prior to their functional characterization is key to better understanding their function. Additionally, standardizing and optimizing the isolation method and providing detailed reporting are essential to ensure more reproducibility between groups, facilitating a better understanding of diverse EV roles.

### Bacterial EV markers and characterization

Mammalian EV characterization includes confirming the presence of luminal and surface EV markers^[122]^. A partial understanding of EV biogenesis and other challenges, such as environmental conditions and their effect on EV cargo, along with the magnitude of species being studied, has so far prevented the community from proposing marker proteins to characterize bacterial EVs^[20]^. Therefore, in the absence of such makers, it is imperative to perform a thorough characterization based on particle size and morphology to ensure successful EV isolation^[121,122]^. Nevertheless, there is a need to perform additional rigorous high throughput omics to study EV cargo, enabling an enhanced understanding of conserved constituents and regulators of biogenesis and secretion^[123]^.

**Figure 4:**
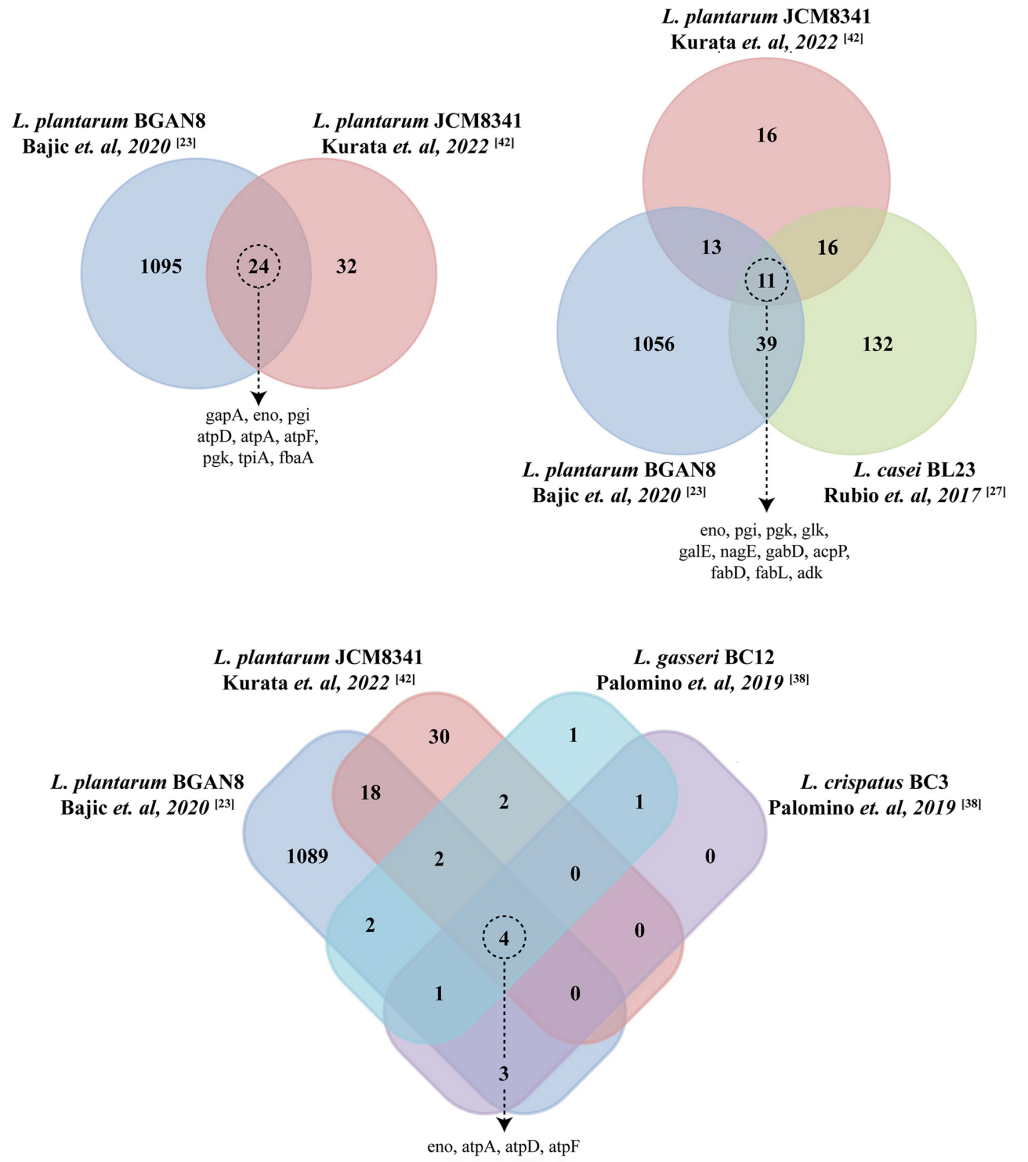
Omics approaches to thoroughly characterize probiotic bacterial EV cargo could aid in the identification of conserved cargo and lead to the identification of EV-enriched proteins conserved between strains and species. Venn diagrams depicting common and unique cargo in EVs of various species and strains of probiotic bacteria. Venn diagrams were generated using FunRich software (version 3.1.4)^[129]^. EVs: Extracellular vesicles.

Collective community efforts may allow for the identification of EV markers for probiotic strains and thus facilitate improved characterization. For instance, comparing protein cargo identified with proteomic analysis of EVs isolated from different species of probiotic bacteria leads to the identification of several conserved protein cargos between species and strains. Interestingly, enolase was one such protein consistently identified in EVs of all the bacterial species compared. Other proteins such as phosphoglycerate kinase, glucose-6-phosphate isomerase, glyceraldehyde-3-phosphate dehydrogenase A, and adenosine triphosphate (ATP) synthase subunits were also conserved between species [Figure 4].

### EV purity

Bacterial cells actively secrete proteins and other factors in their surroundings, and several of these secretory factors could also be shed via EVs^[124,125]^. Consequently, the purity of EV fraction is of prime importance. While standardizing and optimizing methods for EV isolation, careful consideration is required to ensure a final EV preparation devoid of significant soluble or secretory factors. The presence of contaminants could be detrimental to reliable functional characterization^[121]^. For instance, the anti-cancer and apoptotic effect of cell-free supernatants from several probiotic strains has been reported^[126,127]^. More recently, EVs shed by probiotic strains were observed to contribute to this effect^[72-75]^. However, whether the effect is solely due to EVs in the conditioned medium or stems from other factors secreted by bacteria remains unknown. In this regard, *Streptococcus thermophilus* was reported to have a prophylactic effect in colorectal cancer development due to secreted β-galactosidase^[10]^. However, it has also been reported that bacterial EVs preferentially package acidic glycosidases and proteases, including β-galactosidase^[128]^. Thus, in addition to ensuring maximum purity of EV preparation, further experimental controls, such as a conditioned medium devoid of EVs, are needed to confidently assign a functional role to probiotic EVs.

Another aspect of EV purity to consider is to determine the subtype of EV responsible for the observed phenotype *in vitro* and *in vivo*. As previously discussed in the EV biogenesis section, bacterial EV biogenesis may occur through multiple distinct pathways. Therefore, when isolating probiotic EVs, several EV subpopulations may be present in the preparation. Thus, addressing these challenges and limitations with technical advancements is imperative to understand biogenesis pathways and distinguish EV subtypes. This may further enable a thorough understanding of the observed phenotypes and help to attribute them to specific EV subtypes, which is essential for clinical utility.

### Biocompatibility

EV purity is also essential to ensure biocompatibility*.* The presence of immunogenic contaminants may lead to septic reactions upon systemic administration^[130,131]^. Bacterial toxins may have beneficial roles for the host in the organism’s native environment^[132]^. The biodistribution and bioavailability of EVs can change significantly when alternative routes of administration are used^[133,134]^. Thus, several contaminants or even cargo constituents could have a detrimental impact on host physiology as opposed to a beneficial effect observed *in vitro*. This also requires a thorough understanding of the cargo itself and the effects on host physiology prior to administration^[135-138]^. In this regard, engineering the parent bacterium to synthesize EVs devoid of toxic cargo is an attractive alternative^[139]^. However, efforts to knock out a cargo constituent in the parent bacterium could lead to non-related off-target effects, potentially altering EV cargo significantly and leading to a partial or complete loss of desired function^[140]^. Thus, depending on the route of administration of EVs, which impacts their bioavailability, biodistribution, systemic uptake, retention, clearance, and, in turn, their function, addressing the biocompatibility of EVs requires thoughtful evaluation to support their therapeutic utility^[133,134]^.

### Bioavailability and biodistribution

Probiotic EVs are naturally secreted by microbiota in the gastrointestinal tract, and their local uptake and function have been demonstrated widely *in vivo* in their ability to alleviate colitis symptoms and gut dysbiosis and enhance barrier integrity upon oral administration [Table 1]. Mechanisms facilitating uptake and systemic bioavailability of EVs upon oral administration are still poorly understood^[111]^. Recently, transcytosis of probiotic EVs in IECs has been demonstrated^[23,110]^. Further, elevated levels of EVs in circulation following administration of probiotic strain were observed^[102]^. Several studies have even demonstrated systemic function following oral administration of EVs^[78,104]^. However, a limited understanding of the mechanisms involved *in vivo* prevents wider acceptance^[111]^. Furthermore, plausible effects of probiotic EVs due to modulation of gut and systemic immunity and gut microbiota, thus resulting in the systemic phenotype indirectly must be examined^[78,111]^.

## CONCLUSION AND PROPOSALS

Despite their immense potential, several challenges pertaining to the acceptance and utility of probiotic EVs in clinical applications exist. The transition of probiotic EVs from bench to bedside relies on further technical developments in a bid to address the underlying limitations and knowledge gaps. Technical limitations and challenges were enlisted and discussed in the previous sections, along with proposals to address them. Further to these, a major question that remains unanswered is the delineation of EV cargo responsible for the desired effects. Most studies demonstrating the function of probiotic EVs did not link the effect to any particular cargo component [Table 1]. Comprehensive omics approaches are key in delineating the functional EV cargo and need to be prioritized to gain further momentum in this direction^[141]^. While doing so, it is also important to acknowledge that probiotic EVs contain diverse and rich cargos and several constraints remain to delineate the cargos responsible for the observed effects [Figure 3]. For instance, knockout, knockdown, or overexpression of a molecule of interest can have profound effects on EV cargo loading, biogenesis, and even uptake by the recipient, leading to a loss of desired function unrelated to the molecule being examined^[104]^. Lastly, the need to choose appropriate preclinical models is of high priority to advance the progress of the field^[111]^ [Figure 3]. Though the potential of probiotic EVs in clinical applications is immense [Table 1 and Figure 1], several of these studies claimed an effect based on *in vitro* observations. Whether or not the findings would hold true *in vivo* remains unknown. For instance, while the prophylactic effects of probiotic EVs and their potential in treating and sensitizing cancer cells have been observed *in vitro*, these effects still need to be tested *in vivo* using appropriate models and controls^[72-75]^. This would aid in addressing several unanswered questions about probiotic EVs in addition to their therapeutic value such as their ability to selectively target tissues, thus enabling their use as novel adjuvants and drug delivery vehicles for a synergistic effect.

## DECLARATIONS

### Copyright

© The Author(s) 2024.
