# Supplementary material for: Role of probiotic extracellular vesicles in inter-kingdom communication and current technical limitations in advancing their therapeutic utility
Source: Extracell Vesicles Circ Nucl Acids. 2024 Sep 13;5(3):509–26. doi: 10.20517/evcna.2024.39 (PMC11648425; doi:10.20517/evcna.2024.39)
Supplement: Supplementary file 1 [file evcna-5-3-509-SupplementaryMaterials.pdf]

Supplementary Materials

Role of probiotic extracellular vesicles in inter-kingdom communication and current technical limitations in advancing their therapeutic utility

Rahul Sanwlani[#], Kyle Bramich[#], Suresh Mathivanan

Department of Biochemistry and Chemistry, La Trobe Institute for Molecular Science, Melbourne, Victoria 3086, Australia.

[#]Authors contributed equally.

Correspondence to: Prof. Suresh Mathivanan, Department of Biochemistry and Chemistry, La Trobe Institute for Molecular Science, Science Drive, Bundoora 3086, Australia. E-mail: S.Mathivanan@latrobe.edu.au

ANNOTATED BIBLIOGRAPHY [FIGURE 3]

1. Bajic SS, Cañas MA, Tolinacki M, et al. Proteomic profile of extracellular vesicles released by Lactiplantibacillus plantarum BGAN8 and their internalization by non-polarized HT29 cell line. Sci Rep, 2020. 10: p. 21829.

The paper set out to characterised EVs from *L. plantarum* BGAN8, isolated from a stater culture collection and understand how these EVs are internalised to HT29 cells. Then, they compared the proteomic cargo of the whole cell lysate and EV fraction. Following analysis, authors found that the EVs were significantly abundant with proteins predicted to be present in the membrane.

2. Domínguez Rubio AP, Martínez JH, Martínez Casillas DC, Coluccio Leskow F, Piuri M, Pérez OE. Lactobacillus casei BL23 Produces Microvesicles Carrying Proteins That Have Been Associated with Its Probiotic Effect. Front Microbiol, 2017. 8: p. 1783.

Isolation and characterisation of EVs from *L. casei* BL23 strain was performed, and subsequent proteomic analysis was carried out. Authors discovered several proteomic cargo, including p40, p75, and the product of LCABL_31160, suggesting these EVs may play a role in mediating the bacteria-gastrointestinal cell interface.

3. Kurata A, Kiyohara S, Imai T, et al. Characterization of extracellular vesicles from Lactiplantibacillus plantarum. Sci Rep, 2022. 12: p. 13330.

Authors isolated and characterised *L. plantarurm* EVs. They deciphered the protein cargo of EVs. The authors then demonstrated the immunomodulatory effects of probiotic EVs as they led the host immune cells to produce pro and anti-inflammatory cytokines upon recognition by TLR2. N-acylated peptides from lipoprotein19180 (Lp19180) in *L. plantarum* EVs were identified to be responsible cargo in *L. plantarum* EVs.

4. Ñahui Palomino RA, Vanpouille C, Laghi L, et al. Extracellular vesicles from symbiotic vaginal lactobacilli inhibit HIV-1 infection of human tissues. Nat Commun, 2019. 10: p. 5656.

L. crispatus and *L. gasseri* derived EVs were able to prevent infection of vaginal and tonsillar tissues with HIV-1 by preventing attaching of virus particles to host cells, offering protection *ex vivo*.
